# Hydrogels and Wound Healing: Current and Future Prospects

**DOI:** 10.3390/gels10010043

**Published:** 2024-01-05

**Authors:** Varshan Gounden, Moganavelli Singh

**Affiliations:** Nano-Gene and Drug Delivery Laboratory, Discipline of Biochemistry, University of KwaZulu-Natal, Private Bag X54001, Durban 4000, South Africa; 219006317@stu.ukzn.ac.za

**Keywords:** chronic wounds, wound dressings, hydrogels, wound healing, polymers

## Abstract

The care and rehabilitation of acute and chronic wounds have a significant social and economic impact on patients and global health. This burden is primarily due to the adverse effects of infections, prolonged recovery, and the associated treatment costs. Chronic wounds can be treated with a variety of approaches, which include surgery, negative pressure wound therapy, wound dressings, and hyperbaric oxygen therapy. However, each of these strategies has an array of limitations. The existing dry wound dressings lack functionality in promoting wound healing and exacerbating pain by adhering to the wound. Hydrogels, which are commonly polymer-based and swell in water, have been proposed as potential remedies due to their ability to provide a moist environment that facilitates wound healing. Their unique composition enables them to absorb wound exudates, exhibit shape adaptability, and be modified to incorporate active compounds such as growth factors and antibacterial compounds. This review provides an updated discussion of the leading natural and synthetic hydrogels utilized in wound healing, details the latest advancements in hydrogel technology, and explores alternate approaches in this field. Search engines Scopus, PubMed, Science Direct, and Web of Science were utilized to review the advances in hydrogel applications over the last fifteen years.

## 1. Introduction

Wounds have plagued patients for millennia, imposing a substantial burden on their carers, thus earning its designation as the ‘silent epidemic’ [1]. Approximately 4 million cutaneous wounds have been documented to occur annually in affluent countries, with the number in developing nations in ascendance [2]. Skin injury compromises the integrity of the skin’s framework, leading to a wound healing process that is characterized by a well-coordinated series of cellular and molecular reactions that aim to recuperate or replace the injured tissue [3]. Wounds distinguished by synergistic and ordered processes, which lead to uninterrupted wound regeneration, are commonly referred to as ‘acute wounds’. Although minor cutaneous injuries can recuperate, several variables frequently impact wound rehabilitation. These include severe oxidative stress, infection, and underlying medical conditions that result in the development of “chronic or inert wounds” [4]. Chronic wounds exhibit distinctive attributes, which include recurrent infections, a heightened inflammatory phase, and impaired responsiveness of epidermal cells to reparative signals [5]. 

In addition to the impact on psychological, social, and physical health, diminished productivity and high treatment costs impose a financial strain on the healthcare sector, emphasizing the need for efficient wound treatment. The current industry-standard therapies include skin grafts and flaps, dermal substitutes, and skin growth procedures. However, these procedures encounter significant challenges, such as a scarcity of sites for donors and the development of hypertrophied scars, resulting in physiological complications [6]. Hence, there is a dire need for an efficient alternative to overcome the present limitations. 

Hydrogels can be described as intricate three-dimensional structures composed of hydrophilic polymer chains and exhibit a quick swelling response upon contact with water, forming a partially solid material [7]. More than 90% of the hydrogel framework is composed of water, thereby rendering it possible to sustain a moist environment adjacent to the wound’s surface, facilitating tissue repair [4]. Hydrogels possess numerous properties that make them ideal for use as wound dressings. These include firm adhesion, shape adaptability, and mechanical protection, which enable sufficient coverage and safeguarding of the wound [8]. Hydrogel-based dressings possess the advantage of being readily tuneable, allowing for the incorporation of antibacterial and antimicrobial agents, cells, biomolecules, and growth factors [9]. This augmentation aims to expedite the processes of wound contraction and healing. A hydrogel can be constructed using any hydrophilic polymer through a tailored cross-linking technique. These water-soluble polymers can be natural or synthetic. Synthetic materials provide unique features pertaining to their highly modifiable physical attributes and adhesive characteristics. Natural polymers exhibit enhanced biocompatibility and biodegradability compared to synthetic polymers [10]. There has been notable progress in combining natural and synthetic polymers to formulate blended hydrogels. Additionally, integrating nanomaterials in situ has led to the formation of “smart” nanogels that possess a customized functionality, facilitating the application of hydrogels in treating deep or irregular wounds due to in situ induction [11].

Hydrogels represent a subset of therapeutic interventions that have significant promise in enhancing the quality of life for numerous patients affected by wounds and their associated ramifications. This review initially looks at wounds in general, treatment strategies employed, their impact on health and the economy, and the need for suitable therapies. We further present an overview of the advancements made in using hydrogels for wound healing and offer valuable insights into the production of some hydrogel-based wound dressings. Integrating research, cutting-edge technology, and innovative strategies for patient support can provide the impetus needed for the advancement of wound treatments.

## 2. The Skin

**Figure 1:**
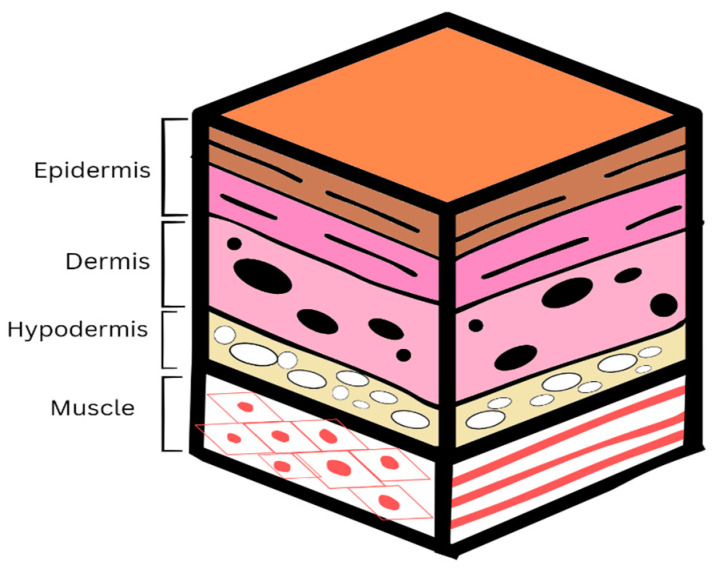
The structure of the skin illustrating the three layers: epidermis, dermis, and hypodermis.

The skin is the most remarkable multifunctional organ in the human body. It plays a crucial role in protecting against a wide range of exterior hazards while preserving the internal environment. In virtue of its physical and sensory functions, this multilayered, complex organ is essential for the body’s defense. Human skin comprises three distinct layers: the epidermis, dermis, and hypodermis (Figure 1). The epidermis, mainly consisting of keratinocytes, is an integral contributor to the skin’s cutaneous protective barrier function [12]. The second layer is the dermis, which is the most substantial stratum of the integumentary system, measuring between 1.5 and 4 mm in thickness. Fibroblasts are the predominant cellular component within the dermis, responsible for the synthesis of collagen and elastin, which contribute to the rigidity and flexibility of the skin. The hypodermis, located beneath the dermis, consists predominantly of adipose and connective tissue, which assists in the provision of strength [13]. Due to the skin’s slight acidity, it is protected against pathogens. In addition, Langerhans cells, which reside within the epidermis, protect against harmful infections [14]. Despite these protective properties, the skin is susceptible to breakage. A wound is characterized by the impairment or disturbance of the body tissue’s anatomical and physiological integrity [15]. Once the disruption has occurred, the skin undergoes a complex and synchronized regeneration process to restore its physical integrity. 

## 3. Wound Healing Phases

**Figure 2:**
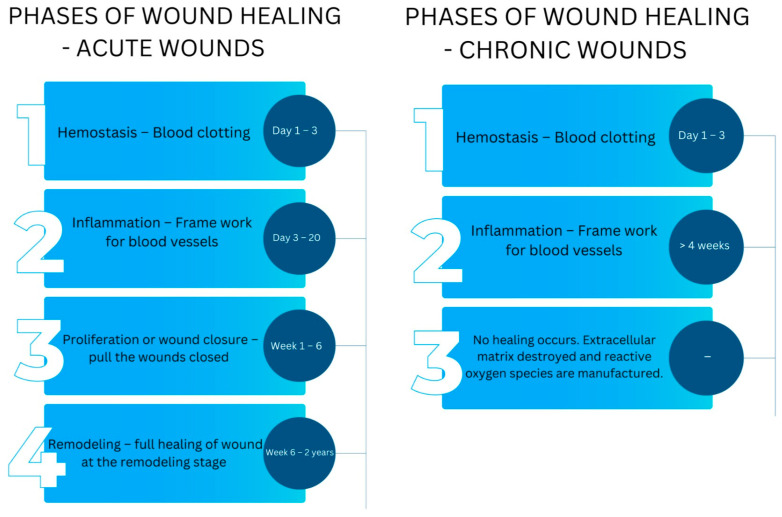
The 4 phases of wound healing and their approximate duration for acute and chronic wounds.

The natural wound repair process is a fundamental physiological mechanism that entails the coordinated interaction of several cellular strains with their respective products [3]. It is essential to maintain the skin’s integrity. To effectively navigate the wound toward complete healing succession, an intricate biological healing process of hemostasis, inflammation, proliferation, and remodeling must be accomplished (Figure 2). 

It is imperative that all four physiological phases, which are closely interconnected, interdependent, and sometimes overlapping, occur in the appropriate sequence and within an acceptable duration [16]. Hemostasis initiates the wound healing process, which occurs when ruptured tissue enables the influx of blood to enter the open lesion. Upon sustaining an injury, the initial physiological response is the restriction of blood vessels, known as vasoconstriction, which impedes the bleeding process [17]. Platelets aggregate upon exposure to collagen and are subsequently released in conjunction with fibrin to form a thrombus, which serves to occlude the punctured blood vessels, preventing bleeding. Additionally, this process provides a provisional structure for the infiltration of cells necessary to repair the lesion [18]. 

Inflammation follows hemostasis, which commences within the initial 72 h post-cellular injury. The cellular response during the period of inflammation is distinguished by the migration of leukocytes into the vicinity of the lesion. A complex cascade of signaling molecules facilitates the influx of neutrophils and macrophages into the region of injury. Neutrophils are mobilized and directed to the wound within the initial 24 h period, where they remain for a duration ranging from 2 to 5 days [19]. These phagocytic cells are responsible for releasing reactive oxygen species (ROS) and lysozymes to eliminate surrounding microorganisms and remove necrotic tissues. Macrophages often migrate to the site of injury within approximately 3 days. They are responsible for the secretion of many growth factors, cytokines facilitating cellular growth, and the formation of molecules within the extracellular matrix (ECM) [17].

The transition from the period of inflammation to the proliferative state is vital in wound healing. The objective of proliferation is to reduce the area of damaged tissue through angiogenesis and fibroplasia, thus producing an effective epithelial screen that can stimulate the activation of keratinocytes for wound closure. These processes commence within an initial 48 h period and may continue until the 14th day following the emergence of injury [3]. The last stage of the healing process involves remodeling, which commences around 2 to 3 weeks following the initiation of the wound and may persist for one year [20]. The primary objective of the remodeling phase is to optimize elasticity and restore the typical tissue structure through reorganization, disintegration, and reconstruction of the matrix surrounding the cells. The granulation tissue undergoes a slow remodeling process, forming scar tissue [21]. Although the process of wound healing is highly efficient, various factors, such as infection and disease, can cause impairment, resulting in an increase in healing time and recurrent wounds.

## 4. Acute and Chronic Wounds

**Figure 3:**
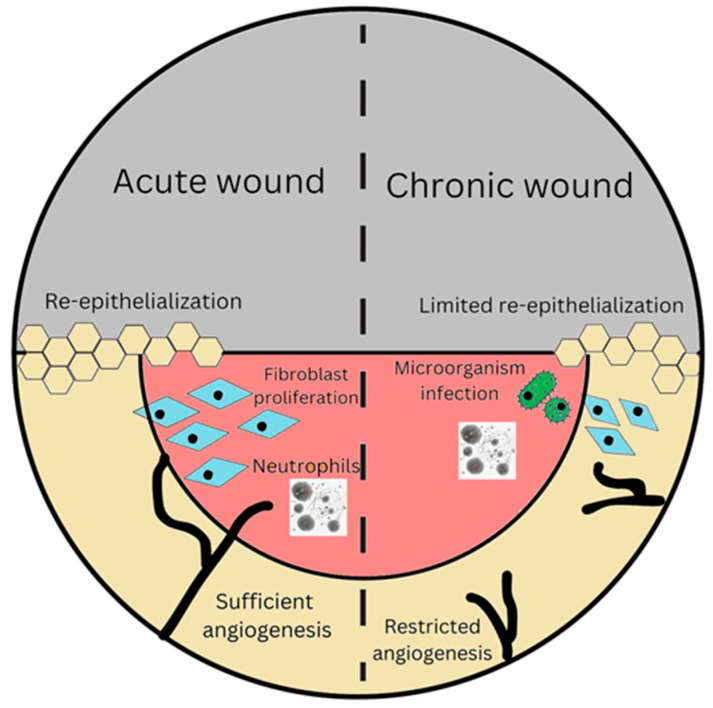
Illustration of the differences in acute and chronic wounds during wound healing.

Acute and chronic wounds are determined based on the healing period after the initial injury and, more significantly, by the presence of physiological damage. Acute wounds are breaches in the epidermis integrity that repair completely, with minor scarring (Figure 3), while progressing through a structured process of recovery that takes about 8 to 12 weeks [6]. The etiology of these injuries primarily stems from mechanical trauma, such as abrasive interaction and incisions performed during surgical procedures, thermal traumas, chemical burns, and electrical accidents [22].

A wound that fails to advance through the standard phases of inflammation and regeneration is classified as a chronic wound. This classification is frequently assigned once the wound exhibits minimal indications of improvement beyond three months [23]. Around 15% of wounds fail to heal within one year following their initial manifestation due to asynchronous wound healing phases [1]. The principal risk factors associated with the development of chronic wounds include age, immunological status, malnutrition, infection, low oxygen levels or perfusion, tobacco use, underlying disorders, drugs, exposure to radiation, and chemotherapy [24]. The primary idea of significance pertains to prolonged hyper-inflammation, a common pathophysiological feature that perpetuates a destabilized environment within a wound, rendering it resistant to the healing process. The dysfunction of macrophages also plays a crucial role in deviating from the normal healing process [25]. 

Bacterial infection is a significant factor in the development of chronic wounds. Chronic wounds are frequently infested by bacteria that create complex communities known as polymicrobial biofilms. Biofilms, when present on a wound, induce a surplus of neutrophils that are unable to engulf the bacteria bound to the biofilm. However, these neutrophils continue to release enzymes (cytokines and proteases) and reactive oxygen species (ROS), which disrupt the migration of cells, hinder wound healing, and compromise the nearby tissue [26]. In cases of long-lasting infections, there is a harmful cycle of continual inflammation induced by ongoing biofilms, resulting in excessive and continuous NETosis, which leads to tissue damage and an increase in biofilm production. The formation and accumulation of dead tissue and exudate serve as a constant source of essential nutrients for the biofilm, allowing it to thrive over the host [27]. The rise in bacterial infections is somewhat attributed to the rise in nosocomial infections post-surgery. Surgical site infections account for 2 million nosocomial infections in the United States, and this is a result of both endogenous flora present in mucous membranes as well as exogenous flora in theatre. The rise in infections ultimately increases the likelihood of chronic wounds [28]. Chronic wounds are commonly classified as vascular ulcers, including venous or arterial ulcers, diabetic ulcers, and pressure ulcers [5].

## 5. Socio-Economic Impact of Chronic Wounds

A large proportion of individuals who endure chronic wounds typically present with concomitant medical disorders, particularly obesity and diabetes. Globally, approximately 463 million individuals have diabetes, with projections indicating a surge to 783 million individuals by 2045 [25]. The prevalence of obesity is relatively proportional, as it is projected that by 2030, obesity will impact over 1 billion adults, constituting approximately one-sixth of the global population [29]. In contrast, the wound healing process is impaired among the senior demographic. Ageing results in a reduction in collagen production, causing the epidermis to regenerate at a decreased rate. By 2050, the global population of individuals aged 60 or above is anticipated to surpass the population of youth aged 10–24, with an estimated count of 2.1 billion compared to 2.0 billion, respectively [30]. The notable rise in diseases and the aging population pose significant concerns regarding the future occurrence of chronic wounds in patients. 

According to research, 70% to 80% of people with wounds receive treatment primarily from community nurses [1]. Health systems must develop more effective ways to cope with the additional workload, which fosters immense and perhaps unsustainable pressure on the already overworked nursing personnel. From an economic perspective, it has been projected that the management of wounds constitutes approximately 3% of the total medical expenditure. In the United States of America, it has been projected that venous ulcers result in the forfeiture of roughly two million working days each year and a projected expenditure of USD 2.5 to 3.5 billion a year for the healthcare system. According to the evaluation, the worldwide wound management industry is anticipated to attain a value of USD 18.7 billion by 2027 [25]. Hence, there is a dire need for more efficient treatments to reduce the social and economic strain placed on individuals and health.

## 6. Current Treatment Methods

### 6.1. Dressings

Various forms of dressings for wounds have been reported (Table 1). However, their suitability differs based on the characteristics of the lesion. The selection of dressing is determined by several variables, including depth, location, size, exudate volume, inflammation, and adhesion to the wound [31]. In comparison to dry dressings, moist dressings possess the capacity to enhance the process of wound repair. Moisture-retentive dressings can protect wounds, reduce the risk of infection, and promote granulation tissue formation. These substances are categorised as gauzes, films, or gels based on their physical characteristics. Dry dressings can cause additional pain and tissue damage as they may adhere to wounds.

### 6.2. Negative Pressure Therapy

Negative pressure wound therapy (NWPT) is a non-invasive therapy that employs regulated negative pressure to generate a vacuum. This vacuum system facilitates the removal of debris and fluid from the site of injury by implementing a closed or foam covering connected to an extraction vessel. This methodology effectively improves the degree of oxygen and moisture in the vicinity of the lesion, thereby facilitating wound repair. NPWT is not recommended in wounds associated with cancer, osteomyelitis, or necrotic tissue in conjunction with eschar. Additionally, NWPT restricts the patient’s range of motion [37].

### 6.3. Surgery 

Surgery includes direct wound closure, skin flaps and grafts, and musculocutaneous flaps. The selection of the surgical intervention is contingent upon various factors, including the precise anatomical site, concurrent medical conditions, and the desired outcomes of the medical procedure [38]. The long-term effects of surgical interventions exhibit variations determined by bed rest and preoperative risk factors. Implementing this procedure necessitates the involvement of medical specialists who possess the expertise required and have access to well-equipped healthcare facilities. In addition, surgery is not a cost-effective method.

### 6.4. Hyperbaric Oxygen Therapy

Patients are administered 100% oxygen within a compression chamber with a maintenance pressure exceeding sea level. The improved degree of oxygen delivery to the wound increases regeneration efficiency, leading to a reduction in the duration required for the healing process [39]. Hyperbaric oxygen therapy (HBOT) is currently employed as a treatment modality for managing non-healing wounds. Implementing HBOT necessitates expensive, specialized equipment and is a time-intensive process. Furthermore, its application is typically restricted to wounds associated with diabetes and pressure-induced ulcers. Although HBOT yields many advantages, it is crucial to acknowledge the notable hazards associated with this treatment. These hazards encompass the possible occurrence of pneumothorax and detrimental effects on the eardrums [37].

## 7. Ideal Wound Healing System

**Figure 4:**
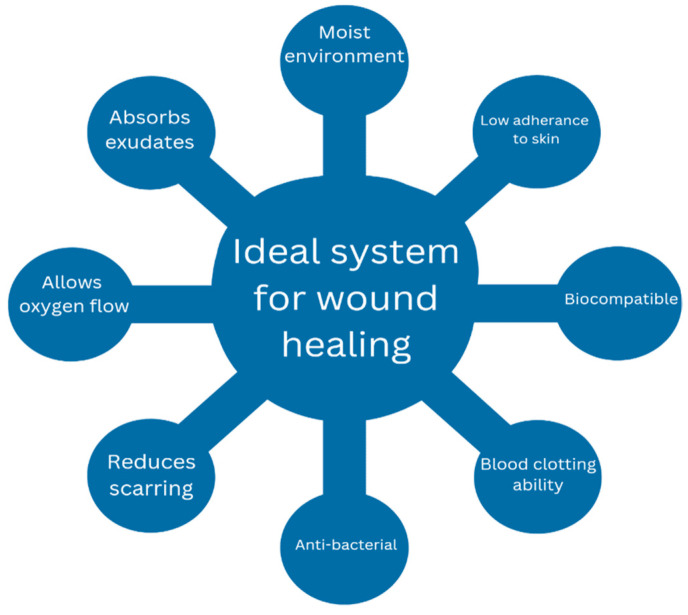
Properties required for an ideal wound healing system.

While numerous conventional wound treatments are available, they exhibit various drawbacks, necessitating the exploration of an alternate optimal treatment strategy. An optimal system should demonstrate antibacterial and antimicrobial characteristics to mitigate infections. During an infection, bacteria infiltrate the site of injury and secrete compounds that impede the ability of immune cells to eliminate these bacteria, thereby prolonging the course of healing [40]. The system must possess biodegradability, biocompatibility, and non-toxicity while also ensuring the provision and maintenance of a moist environment (Figure 4). Moist conditions facilitate the healing procedure by supporting angiogenesis and collagen formation and providing non-adherence, thus reducing pain and scab formation [41]. In addition, the system needs to possess the capacity to absorb wound exudates and facilitate the exchange of gases between the wounded tissue and the surrounding environment. This is crucial, as oxygen is critical in cell growth and angiogenesis [10]. Lastly, the treatment must enhance tissue regeneration mechanisms while demonstrating cost-effectiveness.

## 8. Hydrogels in Wound Healing

**Figure 5:**
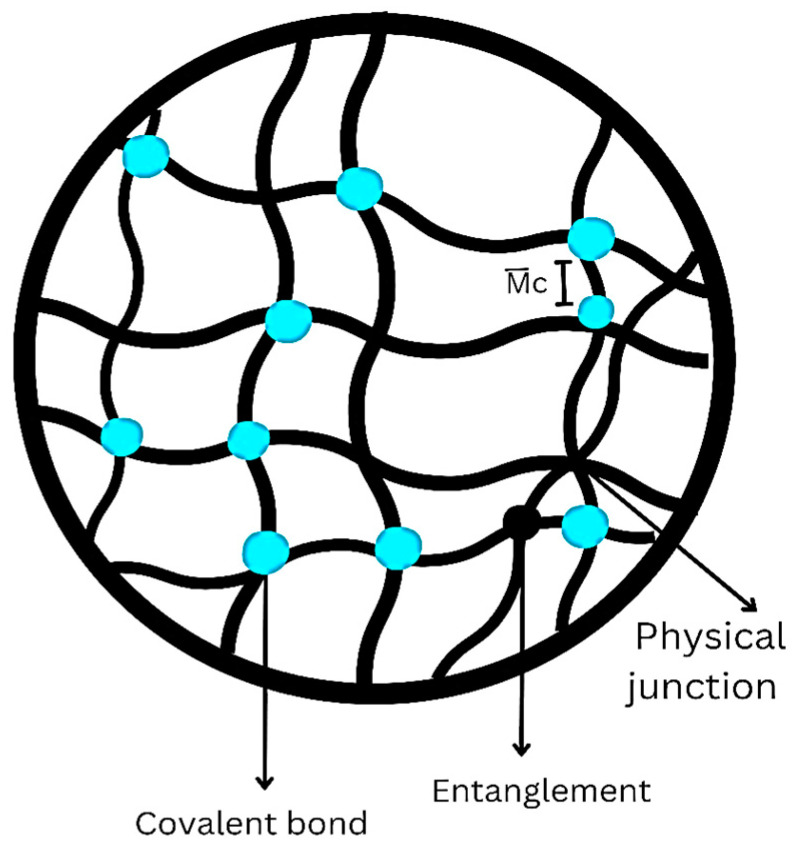
Different types of cross-linking in hydrogels.

Hydrogels are a category of substances that have extensive applications in the field of skin regeneration. Hydrogels are polymeric structures that exist in a three-dimensional structure, formed through physical or chemical cross-linking of hydrophilic polymer chains [42]. They can be synthesized using several techniques, including radiation, freeze–thawing, or chemical processes. Hydrogels are referred to as “reversible” or “physical” gels when the structural integrity is maintained through molecular entanglements or ionic and hydrogen bonds. They are referred to as “permanent” or “chemical” gels when composed of covalent bonds (Figure 5). These networks can potentially undergo water expansion until reaching a state of equilibrium while maintaining their initial structure. This results in a notable capacity to absorb exudates from wounds, facilitate oxygen flow and sustain a heightened moisture content at the injury site. This accelerates the healing process [43]. 

These distinctive physical qualities enable the fabrication of hydrogels into diverse sizes and forms, thereby facilitating the complete covering of irregular-shaped wounds [44]. Hydrogels possess biodegradability and biocompatibility, allowing them to serve as a temporary template throughout the re-epithelialization and remodeling of chronic wounds. Additionally, hydrogels demonstrate sufficient bioadhesivity, which is crucial in ensuring sustained stability. This property enhances hemostasis and maintains optimal moisture levels in the wound [45]. Furthermore, hydrogels provide a versatile framework for incorporating various components such as antibacterial and antimicrobial agents, drugs, and other supplemental biomolecules, enhancing their overall efficacy in promoting wound healing. Hence, it can be concluded that hydrogel-based materials exhibit the highest suitability level as dressings for covering skin wounds.

## 9. Hydrogels as an Extracellular Matrix

Another essential characteristic of hydrogels is their capacity to replicate the extracellular matrix (ECM). The ECM facilitates cellular adhesion, tissue anchorage, cellular signaling, and cell recruitment. The primary components of the ECM include polysaccharides, proteins, and water [46]. In situations of acute or chronic injury, the ECM can be harmed. Hydrogels can imitate the rigidity of an ECM due to their primary elements being water and polymers. Furthermore, hydrogels can imitate the functions of the ECM since they can incorporate cells and other macromolecules found in the ECM [47]. Theoretically, once a hydrogel is placed on the wounded area, it acts as a dermal matrix that is used to replicate the structure and function of unwounded skin, thereby potentially preventing the ensuing scar by approximating the strength of tensile contraction and elastic retraction of an intact, unwounded dermis. This is believed to promote the development of cells, the deposition of the ECM, and the creation of new tissue, thus enhancing wound healing. Hyaluronic acid, collagen, and alginate hydrogels have proven to be particularly valuable for creating ECM matrices [48].

## 10. Hydrogels for Treatment of Burn Wounds

Within the initial 15 min of a thermal burn, the heat generated is stored in the epidermis and then transmitted to the underlying layers. Applying a cooling agent to the skin diminishes the severity of the damage and minimizes scarring [49]. Hydrogels serve a crucial role in burn therapy as primary dressings for first aid. The water in hydrogels provides an essential function in the cooling process and helps maintain a stable temperature in the wound. Hydrogels serve the dual purpose of cooling the burn site and alleviating pain while safeguarding the wound region from infection [50]. Consequently, they are highly suitable as dressings during transportation in ambulances. In comparison to paraffin dressings, hydrogels offer quicker recovery and may be a safe first-aid treatment option for paediatric patients. Many ambulances worldwide are equipped with hydrogel sheets (96% water) for emergencies [51]. Carbomer 940 is an affordable and effective hydrogel used for burns. It has the ability to enhance blood flow to tissues and reduce the extent of necrotic tissue [52].

## 11. Natural and Synthetic Hydrogels

**Figure 6:**
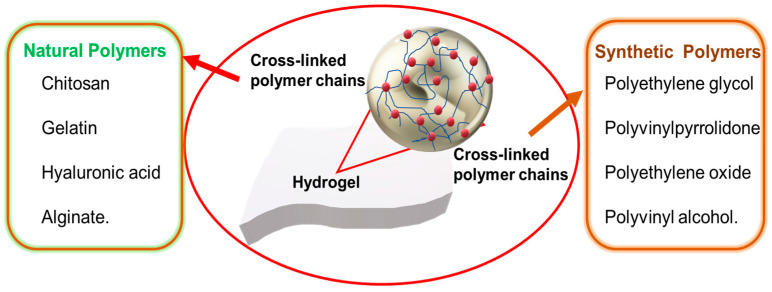
Natural and synthetic polymers being used in hydrogel dressings.

Hydrogel wound dressings are developed using diverse natural and synthetic polymers (Figure 6). Natural polymers include chitosan, gelatin, hyaluronic acid, and alginate. Synthetic polymers include polyethylene glycol, polyvinyl pyrrolidone, polyethylene oxide, and polyvinyl alcohol. Hydrogels can be highly elastic, and this reduces mechanical power; therefore, multipolymeric hydrogels have been introduced for improved mechanical power and absorption. Combining a naturally occurring polymer with a synthetic polymer promises to be a viable approach for generating materials with the desired thermal and mechanical attributes. Advancements in the field have been achieved by harnessing the inherent features of polymers, leading to the development of novel technologies such as sprayable hydrogels, “smart hydrogels”, nanogels, aerogels, and cryogels.

### 11.1. Natural Hydrogels

Natural hydrogels primarily comprise proteins and ECM constituents, rendering them intrinsically biocompatible, bioactive, and potentially well suited for various biomedical applications due to their ability to enhance numerous cellular activities [53]. The makeup and attributes of these materials resemble the inherent characteristics of tissue layers. Nevertheless, they are subject to some restrictions, primarily from the challenges associated with their manipulation arising from variations observed between different batches. Hydrogel variants exhibit unique properties that render them more appropriate for their proposed purpose. We shall briefly discuss the various natural hydrogels.

#### 11.1.1. Chitosan

Chitin is the primary building block of arthropod exoskeletons. It is partially deacetylated to make chitosan, a linear polysaccharide of beta (1-4)-linked D-glucosamine and N-acetyl-D-glucosamine groups. Chitosan exhibits a structural resemblance to glycosaminoglycans inside the ECM. The molecular weight and level of deacetylation of chitosan have a direct relationship with its physical and mechanical characteristics [54]. Chitosan possesses a cationic charge and exhibits specific antimicrobial activity through electrostatic interactions [55]. Its positive charge has made it a polymer of choice for coating nanoparticles for enhanced stability [56,57]. The potential advantages of chitosan-based hydrogels in wound healing applications include the creation of a hydrated wound environment, protection from infections, promotion of leukocyte activity for wound exudate disposal, regulation of degradation through a change in the level of deacetylation, and a reduction in scar tissue [58]. These properties highlight the significant promise of chitosan in wound healing. The susceptibility of the hydrogel to external factors, such as pH and temperature, can be attributed to the presence of hydroxyl and amino groups. One of the drawbacks associated with this material is its suboptimal mechanical strength and challenges in manufacturing fibrous wound dressings [53]. Nevertheless, this issue can be effectively addressed by implementing cross-linking techniques. The most prominent cross-linking technique is the utilization of glutaraldehyde or genipine cross-linkers, which will typically embed themselves between chitosan polymer chains by cross-linking with the amino groups of chitosan. Another method often utilized is the cross-linking of chitosan with tripolyphosphate (TPP). The phosphates present in TPP ionically bind to the amine groups of the chitosan through a process known as ionic gelation [59].

An injectable chitosan-based hydrogel for repairing wounds has been reported. This hydrogel exhibited antimicrobial activity against bacterial strains *Pseudomonas aeruginosa* and *Staphylococcus aureus*, with a terminating efficiency of 96.4% and 95.0%, respectively. Hydrogel-treated wounds showed 99.8% sealing after two weeks. Evaluation of the hydrogel’s hemostatic properties demonstrated prompt attachment to the adjacent tissue of the bleeding region, thereby establishing a protective covering that mitigated hemorrhaging [60]. Hence, hydrogels derived from chitosan can promote the healing of wounds as well as the prevention of infection. Recently, an injectable carboxymethyl chitosan (CMCS) hydrogel to modulate cellular responses and facilitate the complete recovery of diabetic wounds was developed. CMCS was synthesized by modifying chitosan to improve its solubility in water. The CMCS hydrogel exhibited a significant swelling rate of 132% at 37 °C. This property enabled it to efficiently soak up a substantial quantity of tissue exudate and regulate the moisture levels at the lesion. The hydrogel was administered intradermally into the wounds of mice with diabetes. The hydrogel promptly attached to the location of the wound, effectively halting hemorrhaging and establishing a favorable environment for the healing process, which took 14 days for 99% wound healing [61]. Hence, it can be concluded that hydrogels constructed from carboxymethyl chitosan exhibit properties that closely resemble the conditions of the ECM, demonstrating their efficacy in wound healing. 

#### 11.1.2. Gelatin 

Gelatin is a renowned, naturally occurring, inexpensive vascular polymer with beneficial features for tissue development, including low immunogenicity and significant degradability. Gelatin is derived by disrupting the triple helical structure of collagen, resulting in the development of the sequence of amino acids known as RGD (Arg/Gly/Asp). This sequence can facilitate the adhesion of cells and create a favorable environment for cell proliferation. This material’s fibroblast adhesion, proliferative features, and low antigenicity render it highly promising for clinical use [62]. Nevertheless, its suboptimal strength and susceptibility to breakage constrain its utility as a hydrogel treatment. Therefore, this hydrophilic protein requires cross-linking [63]. Gelatin has also been employed as an adhesive for coating to enhance cell adhesion, serving as a method for facilitating the regrowth of vascular tissues. The absorption of wound exudates and moisture maintenance by porous gelatin matrices contribute to the facilitation of wound recovery. Despite its potential as a biopolymer for wound healing applications, gelatin lacks antibacterial properties that could effectively avoid infections. Therefore, it is typically combined with antibacterial agents or hybrid polymers [64]. Gelatin is often cross-linked with aldehydes, such as glutaraldehyde and formaldehyde, similar to chitosan, due to the presence of amino groups. An alternative method is to use cross-linkers such as 1-ethyl-3-(3-dimethylaminopropyl) carbodiimide hydrochloride (EDC), that are not incorporated into the gelatin matrix. EDC activates the carboxyl groups present in gelatin, which then undergoes a direct reaction, forming bonds with the adjacent amino groups [65].

Wang and coworkers (2023) developed a bilayer gelatin hydrogel with photothermal properties to eradicate biofilms and provide extensive therapy for chronic wounds. To enhance the attachment and growth of fibroblasts, gelatin methacryloyl (GelMA) with favorable rigidity was synthesized via optical cross-linking on the outermost layer of the hydrogel. In the interim, epidermal growth factor (EGF) was introduced into GelMA to enhance tissue repair and re-establish the wound’s epithelial layer. Scanning electron microscopy (SEM) revealed a significant reduction in the biofilm layer within the lesion after photothermal therapy. Following a 12-day treatment period, the *Escherichia coli*-afflicted wound exhibited a reduction to a mere 7.9% of its initial area [66].

#### 11.1.3. Hyaluronic Acid

Hyaluronic acid (HA) is a glycosaminoglycan lacking sulfonate groups and is a naturally occurring anionic polysaccharide. It comprises a series of disaccharides, specifically β-D-glucuronic acid and N-acetyl-D-glucosamine, interlinked by alternate β-1, 3, and β-1, 4-glucosidic linkages. HA is found within the vitreous humor of humans, umbilical cords, and connective tissues and is synthesized via fermentation by microbes [67]. HA does not undergo hydrogel formation through conventional physical cross-linking methods. However, it is worth noting that this polymer can undergo chemical modifications in its hydroxyl and carboxyl moieties. As a result, it is widely used in the construction of hydrogels, making it one of the most commonly employed polymers. The hydrophilicity of hyaluronic acid can be attributed to the presence of these functional groups, and this characteristic allows it to soak up exudate and enhance cell adhesion efficiently [68]. A widely used cross-linking method for HA is through the formation of thiol-modified HA hydrogels. This cross-linking system entails the conjunction of oxidised glutathione with an HA-based hydrogel through a thiol-disulfide exchange reaction. The thioether–sulfone bond is highly stable and not susceptible to hydrolysis, making it suitable for hydrogel formation [69]. Hydrogel wound dressings based on HA exhibit notable features that make them a good option for addressing all four phases of wound healing. These characteristics encompass a reduction in inflammation, an amplification of angiogenesis, and the promotion of endothelial cell growth [70].

Li et al. (2022) developed a hydrogel that used HA as its primary constituent. HA was cross-linked using benzaldehyde-functionalized PEG co-polyglycerol caprate (PEGSB) to form a hydrogel with both elasticity and regeneration abilities. The aldehyde group of PEGSB can undergo a chemical reaction with those found in the wounds, thereby facilitating adequate adhesion. A three-minute exposure to near-infrared (NIR) irradiation at 808 nm effectively eradicated *Escherichia coli* and methicillin-resistant *Staphylococcus aureus* (MRSA). After a 14-day therapy regimen targeting hip wounds in mice, a near-complete healing of the lesion was observed. The hydrogel showed promise for practical use in the treatment of infections in wounds due to its excellent biocompatibility [71].

#### 11.1.4. Alginate

Alginate is a polymer found in brown algae cell walls and certain bacteria capsules. Its structure is built from blocks of two distinct monomers, D-manuronate (M) and L-glucuronate (G). Alginate’s substantial G block concentration may produce stiff hydrogels when bound to divalent cations like Ca^2+^. This is referred to as the “egg-box model”. Alginate with an elevated M block concentration demonstrates reduced adhesiveness and immunostimulatory properties [72]. The calcium ions in an alginate-containing dressing exchange with sodium ions as they come into contact with the exudate from the wound. The alginate fibres undergo expansion, partial dissolution, and solidification, forming a protective coating that facilitates wound repair [73]. A direct method of cross-linking is typically utilized, where alginate is directly treated with calcium chloride, calcium sulphate, or calcium carbonate. Internal gelation occurs upon mixing as the cations penetrate the alginate gel through diffusion. Divalent ionic bonding with zinc oxide has also been a notable cross-linking method, as it provides antibacterial properties [74]. Alginate can activate macrophages and promote the production of interleukin-6 (IL-6) and tumor necrosis factor α (TNF-α) by monocytes, thereby accelerating chronic wound healing. Dry alginate dressings can soak up wound fluids, resulting in the formation of gels. These gels subsequently release water, which can benefit the hydration of dry wounds. The gelation property of alginate facilitates the painless and safe removal of dressings [75]. Multiple commercial hydrogels have alginate as the primary component (Table 2).

To improve the repair of wounds, a dual-network hydrogel using platelet-rich plasma (PRP) and sodium alginate (SA) was synthesized using a thrombin activation method. The presence of epidermal growth factor (EGF) and vascular endothelial growth factor (VEGF) was observed in a hydrogel maintained in phosphate-buffered saline (PBS), suggesting the potential for cellular growth and the redevelopment of blood vessels. The hydrogel showed efficacy in promoting wound closure when administered directly to the cutaneous wounds of rats [76]. Using alginate hydrogels presents a viable strategy for addressing the limitations associated with traditional wound dressings. 

### 11.2. Synthetic Hydrogels

Synthetic polymers have demonstrated considerable efficacy in biomedical applications due to their mechanical properties, capacity for facile shaping into various configurations, and manufacturing cost-effectiveness [77]. These polymers exhibit stability and ease of use but are inhibited by limited biocompatibility. In contrast to their naturally occurring equivalents, synthetic polymers possess the advantage of being conveniently manufacturable on an industrial level. Furthermore, their inherent adaptability enables them to be employed in various forms that promote the ideal development of tissues. The ability to precisely manipulate both the hydrophilic and hydrophobic regions of synthetic polymers additionally permits the fabrication of more homogenous frameworks and an improved capacity for the retention of water [78]. Hybrid polymers (blended), which exhibit favorable physicochemical characteristics, can be achieved through their combination with biopolymers. The positive attributes of blended hydrogels fabricated from synthetic polymers are further improved using bioactive substances derived from naturally occurring substances. Blended hydrogels serve as a solution for future wound treatment by combining their favorable characteristics. Some synthetic polymers include polyvinyl alcohol, polyethylene glycol, and polyvinylpyrrolidone.

#### 11.2.1. Polyethylene Glycol (PEG) 

Polyethylene glycol (PEG) is a polymer that possesses hydrophilic properties, resulting in it being capable of interacting favorably with water. PEG is characterized by its flexibility and is composed of ether-based units. 

The use of PEG-based hydrogels in constructing biological systems has been motivated by their remarkable biocompatibility and ability to thwart protein attachment. The addition of functional groups may generate PEG derivatives like PEG dimethacrylate (PEGDM) and PEG diacrylate (PEGDA), which can then be chemically cross-linked to create long-lasting matrices that permit the connecting or integrating of biomolecules to support tissue repair [78]. Polymethacrylic acid (PMA) and polyacrylic acid (PAA) can combine with PEG to create complexes by hydrogen bonding between the carboxyl groups of PMA and the oxygen of PEG. This facilitates the absorption of liquids by the complex, causing it to expand at low pH, forming a gel. PEG may also be used as a cross-linker due to its rigidity, water solubility, and low immunogenicity [79]. Growth factors, such as epidermal growth factor (EGF) and PEG macromers, have a favorable attraction and can form chemical bonds with each other. These can be specifically directed to the site of injury. PEG’s mechanical, thermal, and crystallinity attributes can be enhanced by including chitosan in the polymer blends [80]. PEG-based hydrogels have been used for the treatment of lesions in individuals with diabetes. These hydrogels facilitate wound repair by stimulating the multiplication and development of skin cells. The application of such dressings has been observed to decrease scar development.

A wound closure study using PEG-based hydrogels on 1.5 cm long incisions in Sprague Dawley rats was conducted by Chen et al. (2018). Applying the PEG-based hydrogels stopped the hemorrhaging from the cuts and the incision apertures closure of the incision within minutes [81]. Hence, the use of PEG-based hydrogels can exhibit a positive impact on the wound healing process. 

#### 11.2.2. Polyvinyl Alcohol (PVA)

Polyvinyl alcohol (PVA) is a hydrophilic polymer featuring properties that have garnered considerable attention from the biomedical industry. It is biocompatible, biodegradable, and semi-crystalline. 

PVA can undergo physical cross-linking using several freeze–thaw cycles, called cryogelation. Additionally, PVA can be chemically cross-linked by employing glutaraldehyde or epichlorohydrin. Both methods of production produce PVA hydrogels that are remarkably hydrophilic and chemically stable [82]. PVA can be altered with glycidyl methacrylate or acryloyl chloride to produce reactive acrylate groups via the pendant hydroxyl groups. These can then be cross-linked and polymerized to create hydrogels [79]. PVA hydrogels serve as effective wound dressings by protecting them from external environmental stimuli and mechanical forces, reducing the risk of secondary injuries. Furthermore, PVA hydrogels exhibit favorable characteristics such as excellent water and oxygen permeability and an elevated moisture level [83]. These attributes are particularly advantageous in wound healing, as they facilitate maintaining a moist environment, promoting the formation of new tissue, and improving the overall wound healing process. However, PVA hydrogels lack inherent antibacterial activity, necessitating the augmentation of their antibacterial efficacy when employed as a therapeutic [84].

Through the coupling of chitosan/Fe^3+^ and carboxylated polyvinyl alcohol, a double-cross-linked hydrogel was synthesized that exhibited exceptional features, including enhanced rigidity (78 kPa) and adherence traits, as well as a reduced duration for self-healing (5 min). These changes were observed to align with the dynamic nature of lesions. The hydrogel demonstrated antibacterial efficacy and enhanced hemostatic ability throughout the wound recovery phase. Furthermore, it was proposed that the hydrogel could reduce skin repair duration to 14 days [85].

#### 11.2.3. Polyvinylpyrrolidone (PVP)

Polyvinylpyrrolidone (PVP) is a crystalline polymer soluble in water and polar solutions. PVP is a highly appealing polymer for the manufacture of hydrogels due to its diversified qualities, including its non-toxic nature, ability to form films, and adequate adhesion. A promising technique for the cross-linking of PVP is the use of radiation. PVP is often mixed with PEG and agar to form a reaction mixture that undergoes cross-linking through irradiation under a linear electron accelerator. Radiation cross-linking removes the need for an initiator and cross-linking agent and allows for easy manipulation of the hydrogel properties [86]. PVP can absorb water up to a magnitude of one hundred times its mass, which facilitates the preservation of moisture [87]. The semi-permeable nature of PVP enables the selective permeation of oxygen while effectively impeding the ingress of bacteria and other contaminants. The utilization of PVP is beneficial in the debridement process, as it effectively absorbs exudate and necrotic tissue. PVP can potentially protect wounds from further injury or the onset of infection [88].

Hydrogel fibre mats using PVP with ferulic and p-coumaric acid have been synthesized. The biocompatibility studies conducted on erythrocytes from humans, A549 cells, and HaCaT cells demonstrated the absence of any adverse impacts. The ex vivo experiments on human skin revealed evidence of skin regrowth and effective regulation of inflammation, as seen by the presence of minimal quantities of pro-inflammatory cytokines, specifically IL-6 and IL-8. Hence, the results obtained from the study suggest that PVP-based fibre hydrogels have the potential for application in wound treatment [89].

## 12. Advanced Hydrogels

### 12.1. Sprayable Hydrogels

Attempts have been made to address the shortcomings of traditional dressings used in healthcare by employing “in situ” forming wound dressings such as sprayable hydrogels. These hydrogels provide many benefits, including ease of use without needing specialized assistance, patient approval, and manufacturing cost-effectiveness [6]. Furthermore, applying a spray can enhance the permeation of the hydrogel into the injured region, improving the administration of medicinal formulations or active substances. An ideal viscosity is thus needed to allow the sprayability of these hydrogels [90]. 

A methacrylate gelatin (GelMA) hydrogel is an example of a sprayable hydrogel. Cheng and coworkers (2021) functionalized the GelMA hydrogels with DOPA, resulting in an increased affinity for attachment to wound surfaces. The GelMA-DOPA hydrogel was also loaded with cerium oxide nanoparticles (CeONs) and an antimicrobial peptide (AMP HHC-36) to grant the hydrogel antibacterial and ROS-scavenging properties. This hydrogel exhibited several advantageous properties, including sprayability, adequate adhesion, antibacterial activity, ROS-scavenging, and wound repair capabilities. These attributes have the potential to effectively alleviate the medical and financial obstacles related to the care and handling of chronic wounds [91]. A few constraints associated with sprayable hydrogels include the control of drug dispersity and uniform spraying, balancing rheological properties while maintaining physical and mechanical characteristics, and the inhibition of precursors during gel formation by bioactive molecules [92].

### 12.2. “Smart” Hydrogels

**Figure 7:**
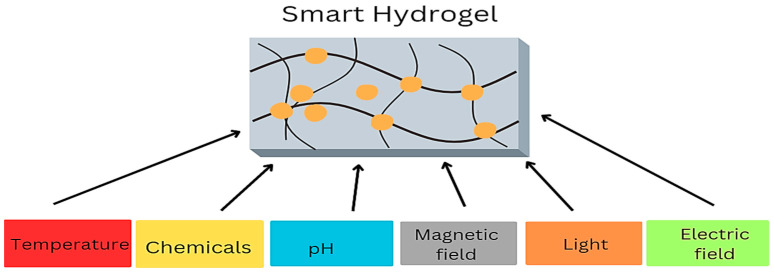
Sensors detected by “smart” hydrogels.

Conventional wound therapies cannot offer insights into the overall progress of healing. This served as a catalyst for advancing sensor-based hydrogels, commonly called “smart” hydrogels. These hydrogels can present significant insights into the state of a wound, encompassing factors such as the concentration of bacteria, oxygen levels, inflammatory intensity, pH, and temperature (Figure 7). Many sensors have been created to quantify the pH level, moisture, temperature, oxygen supply, and mechanical and enzymatic activity [6]. Crucially, each sensor must be non-toxic, biocompatible, and flexible enough to conform to the hydrogel. Temperature has been identified as a handy parameter for the early identification of infection within the wound, as aberrant fluctuations in wound temperature can serve as an initial indicator of an infection before the manifestation of any other symptoms [93]. However, there are concerns associated with thermosensitive hydrogels, such as weak mechanical properties, poor biocompatibility, and a delayed temperature response [94].

A “smart” hydrogel consisting of a polycation and alginate was formulated and demonstrated remarkable antibacterial properties against *Staphylococcus aureus* and *Escherichia coli*. Treatment with this hydrogel promoted the healing of infected wounds in rats, with a recovery rate of 96.49%. The hydrogel also reacted to stress, temperature, and pressure, demonstrating its potential for reliable wound monitoring [95].

## 13. Alternative Gels for Wound Healing

### 13.1. Nanogels

**Figure 8:**
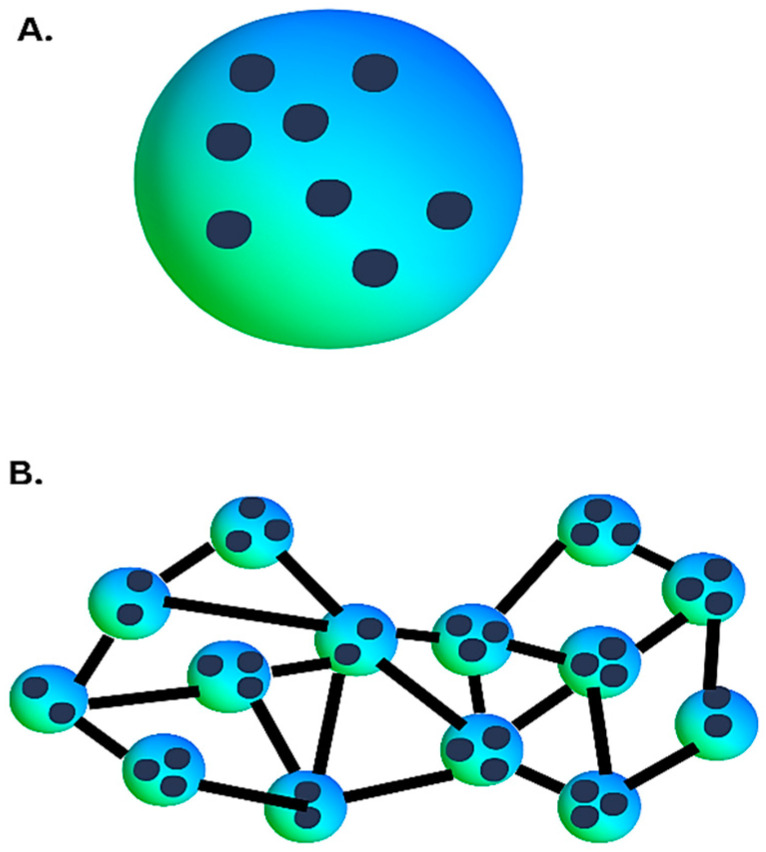
(**A**) Nanoparticles embedded in a hydrogel. (**B**) Cross-linking between polymer particles.

Nanogels are hydrogels composed of cross-linked polymer networks in three-dimensional configurations at the nanoscale. The nanogels primarily exhibit a spherical shape and can be constructed to possess a core–shell or core–shell–corona structure (Figure 8). 

Nanogels have garnered significant interest due to their ability to integrate the characteristics of hydrogels, such as substantial water retention and adaptable physical characteristics, with the customizable size and expansive surface area of nanoparticles for the conjugation of active compounds [96]. Nanogels that demonstrate a response to stimuli enable the controlled release of therapeutic agents (antibacterial agents, cytokines, and growth factors) in response to illness-induced fluctuations in pH and temperature, which are commonly associated with infection. Additionally, nanogels have the potential to significantly contribute to the precise adjustment of the texture of the scaffold and its mechanical features, which is crucial for cell regulation [97].

Metal nanocomposite gels have recently gained attention as promising nanogels for enhancing wound healing while serving as a shield against bacterial infections. The nanomaterial’s moist state can effectively inhibit wound dehydration, which impedes the wound recovery process. Simultaneously, the nanoparticles incorporated inside the hydrogels can function as antibacterial or antifungal agents by hindering the development and proliferation of bacteria or fungi [98]. Haseeb and colleagues synthesised linseed hydrogels (LSH) containing silver nanoparticles (AgNPs). The study revealed that the LSH-AgNPs effectively suppressed the proliferation of bacteria and fungi. Furthermore, 100% wound closure in rabbits was exhibited on day 15, demonstrating its therapeutic efficacy [99]. Some issues halting the progression of nanogels include degradation and batch-to-batch reproducibility [96]. 

### 13.2. Aerogels

Aerogels are characterized as substances consisting of over 99% air. They can be synthesized from various precursors (inorganic and organic) and are frequently fabricated in diverse configurations to fulfill specific requirements. Aerogels exhibit a lightweight composition with a linked pore network [100]. This structure is achieved by replacing the liquid within the gel with a gaseous substance. These dressings present notable benefits, such as low density, high porosity, and a large surface area. Due to these inherent characteristics, aerogels facilitate the rapid intake of a substantial volume of exudate, sustain a hydrated environment, enable gas diffusion, and concomitantly provide commendable thermal insulation for maintaining physiological temperature [2]. Although aerogels show promise for utilization in wound treatment, challenges such as characterization of optical and thermal characteristics of a single aerogel fibre, manufacturing single fibre devices, and scalability need to be overcome [101].

### 13.3. Cryogels

Cryogels are macroporous hydrogels that can possess shape memory function and have the potential for use in wound healing and hemostasis [102,103]. Due to their structure, cryogels can promote the adherence of platelets, which further improves hemostasis [104]. In a recent study, a cryogel was formulated by cross-linking chitosan with citric acid at very low temperatures. Silver nanoparticles produced by tannic acid reduction were incorporated into the cryogel for added antibacterial properties. The produced cryogel possessed an interconnected macroporous network, good mechanical features, hemostasis, shape memory, biocompatibility, antibacterial activity, and promoted wound healing in rats [105]. Hence, cryogels may be a potential strategy for obtaining an innovative wound treatment method. Some practical constraints include scalability, mass transfer, and structural integrity [106].

## 14. Conclusions and Future Perspectives

The predicament of chronic and acute wounds has impeded the social and economic climate, and much-needed change in healthcare is required to rectify these detrimental effects. The current strategies in wound healing have multiple shortcomings in reducing recovery duration. Hence, a new and improved system is needed to overcome these incompetencies. Hydrogels have been presented as adequate wound dressings for chronic and acute wounds through their ideal properties, such as maintaining a moist environment, absorption of exudates and necrotic tissue, and flexibility in shape to cover wounds with different morphologies. Hydrogels consist of natural or synthetic polymers, with a combination of the two being ideal for forming blended hydrogels that possess favorable traits for wound healing. The next generation of wound dressings entails sprayable hydrogels, allowing efficient covering of wounds in any shape or form. “Smart” hydrogels have gained prominence due to their ability to monitor the state of the wound using sensors. The development of innovative hydrogels can be a solution for reducing the wound healing period, improving overall patient health and satisfaction, and reducing the load borne by the current healthcare system. The advent of “smarter” hydrogels is a possibility.

## Figures and Tables

**Table 1:** Common wound dressings.

Type of Wound	Treatment	Function	Advantages	Disadvantages	References
Infected	Gauze	Dries the wound	Removes necrotic tissue; used with topical products; can pack wounds	Adherence hinders healing. Frequent change in dressing needed. Secondary dressing necessary	[32,33,34]
High exudate	Foam	Absorbs high levels of exudates	Provides a moist environment. Easy to apply. Non-adherent	Adherence hinders healing. Unsuitable for eschar/non-draining wounds	[32,33,35]
Superficial skin disruption	Film	Allows for exchange of gases.	Stabilizes the wound site. Easy to visualize. Autolytic debridement	Damages new tissue. Poor moisture absorbance. Periwound maceration.	[32,33,36]
Eschar	Hydro- colloid	Absorbs high levels of exudates	Provides a moist environment. Insulation. Autolytic debridement. Is waterproof	Promotes granulated tissue. Unsuitable for infected wounds.	[25,32,33]

**Table 2:** Some commercially available hydrogels for wound healing [5].

Product	Company	Constituent	Use
DermaSyn^®^	DermaRite Industries (NJ, USA)	Primary wound dressing with vitamin E	Partial and full-thickness chronic wounds
Neoheal^®^ Hydrogel	Kikgel	Polyethylene glycol, polyvinylpyrrolidone, Agar, and 90% water	Low-exuding scabs, a abrasions, dry scabs, first, second-and third-degree burns, and ulcers
Restore Hydrogel	Hollister Inc. (IL, USA)	Gauze pad, Hyaluronic acid	Partial and full-thickness chronic wounds
ActivHeal^®^	Advanced Medical Solutions Ltd. (Oxon, UK)	Primary wound dressing with 85% water	Cavity wounds, pressure ulcers, diabetic foot ulcers, and leg ulcers
NU-GEL™	Systagenix	Sodium alginate primary wound dressing	Diabetic foot ulcers, leg ulcers, venous ulcers
Purilon^®^	Coloplast	Calcium alginate, sodium carboxymethyl cellulose	Pressure ulcers, first and second degree burns, non-infected diabetic foot ulcers, leg ulcers
Simpurity™ Hydrogel	Safe n’ Simple	Acrylate, polyvinyl alcohol, polyethylene oxide, polyurethane	First and second-degree partial- thickness burns, low-exuding chronic wounds

## Data Availability

Not applicable.
